# Utilizing sponge spicules in taxonomic, ecological and environmental reconstructions: a review

**DOI:** 10.7717/peerj.10601

**Published:** 2020-12-18

**Authors:** Magdalena Łukowiak

**Affiliations:** Department of Environmental Paleobiology, Institute of Paleobiology, Polish Academy of Sciences, Warsaw, Poland

**Keywords:** Porifera, Sponge spicules, Spicular analysis, Paleoenvironment, Marine and freshwater chemistry, Silica fractionation, Silicon isotopes

## Abstract

Most sponges produce skeletons formed by spicules, structural elements that develop in a wide variety of sizes and tridimensional shapes. The morphologies of spicules are often unique to clade- or even species-level taxa which makes them particularly useful in taxonomic assignments. When dead sponge bodies disintegrate, spicules become incorporated into sediments and sometimes accumulate into enormous agglomerations called spicule mats or beds, or fossilize to form special type of rocks called the spiculites. The record of fossil and subfossil sponge spicules is extraordinarily rich and often serves as a basis for far-reaching reconstructions of sponge communities, though spicules are also bearers of significant ecological and environmental information. Specific requirements and preferences of sponges can be used to interpret the environment in which they lived, and reconstruct oscillations in water depths, pH, temperatures, and other parameters, providing snapshots of past climate conditions. In turn, the silicon isotope compositions in spicules (δ^30^Si) are being increasingly often used to estimate the level of silicic acid in the marine settings throughout the geological history, which enables to reconstruct the past silica cycle and ocean circulation. This contribution provides a review of the use of sponge spicules in reconstructions of sponge communities, their ecology, and environments, and aims to detect the pertinent gaps in their utilization. Even though spicules are well known for their significance as bearers of taxonomic, ecological, and environmental data, their potential remains to be fully exploited.

## Introduction

Sponges (Porifera) are a species-rich clade of the earliest-diverging metazoans (Wörheide et al., 2012), with a global distribution (van Soest et al., 2012), diverse ecologies and functions (Wulff, 1984, 2006; Bell, 2008; Maldonado, Ribes & Van Duyl, 2012; De Goeij et al., 2013; Goeij, Lesser & Pawlik, 2017), and a record spanning at least the entire Phanerozoic (Reitner & Wörheide, 2002). Among the four sub-clades of Porifera, three (Demospongiae, Hexactinellida, and Homoscleromorpha) produce skeletons of amorphous silica (Hooper & Van Soest, 2002) and one (Calcarea) of magnesium-calcite (Rossi et al., 2014). These skeletons are composed of elements called spicules (Uriz et al., 2003; Sethmann & Wörheide, 2008).

Spicules provide structural support for maintaining the vertical body position, minimize the metabolic cost of water exchange (Riisgard & Larsen, 1995; Uriz et al., 2003), and may even deter predators (Uriz et al., 2003 and the literature cited therein). They often develop in different sizes (Hooper & Van Soest, 2002) and a wide variety of tridimensional shapes, with many being unique to clade- or even species-level taxa. Demosponges are characterized by spicules of monaxonic or tetraxonic symmetry (Hooper & Van Soest, 2002). Hexactinellids produce spicules of hexactinic or triaxonic (cubic) symmetry or shapes that are clearly derived from such morpohologies (Leys, Mackie & Reiswig, 2007). The spicules of homoscleromorphs represent peculiar tetractines (calthrops) and their derivatives that originate through reduction or ramification of the clads (Muricy & Díaz, 2002). Spicules of Calcarea are produced in three basic forms: diactines, triactines and tetractines (Manuel et al., 2002).

The mineral composition of sponge spicules makes these structures the most resistant parts of the sponge bodies (Uriz, 2006) and ensures the ability of spicules to withstand various taphonomic processes (Rützler & Macintyre, 1978; Łukowiak, Pisera & O’Dea, 2013), resulting in that they often constitute the only evidence of the presence of some sponges in an ecosystem (Łukowiak, 2016b). Even though sponges are often known from rich assemblages of bodily-preserved specimens (Volkmer-Ribeiro & Reitner, 1991; Olszewska-Nejbert & Świerczewska-Gładysz, 2013; Łukowiak & Pisera, 2016), a significant part of their fossil and subfossil record is also represented by their spicules. Having that in mind, spicules can be of crucial importance for reconstructions of extinct or cryptic (hiding in cervices and caves) sponge communities; and, indeed, they have been investigated especially with respect to their taxonomic significance (Díaz & Rützler, 2001; Hooper & Van Soest, 2002). The morphologies of spicules and their arrangement, together with other important sponge features, such as the shape, consistency, and color, are essential when identifying sponges (Collin et al., 2005).

In contrast to whole-bodied sponge fossils, spicules are common in many depositional environments (Pisera, 2006). Their significance, however, is often underestimated, which is mostly due to the difficulties in assigning disassociated spicules to sponge taxa or due to the scarcity of the material.

Despite that numerous studies reviewed the current knowledge of various neontological (Manconi & Pronzato, 2015) and paleontological (Pisera, 1999) aspects of sponges, including their importance for evolutionary, ecological, and environmental reconstructions (Harrison, 1988b; Pisera, 2004, 2006; Sim-Smith, Ellwood & Kelly, 2017; De Freitas Oliveira, Da Costa & Benedito, 2020), spicules alone—though discussed to some degree in all these studies—were given considerably less attention (Cohen, 2003; Pronzato, Pisera & Manconi, 2017). For instance, Harrison (1988a) and Frost (2001) discussed the utilization of freshwater sponge spicules in paleolimnological studies. Other than that, however, sponge spicules have never been the subject of a detailed review that would summarize their utility in paleontological and neontological studies.

The purpose of the present contribution is to review the use of loose sponge spicules (that is, those disassociated with sponge bodies), a widespread component in fossil, subfossil, and recent marine and freshwater settings, in reconstructions of extinct and modern sponge communities, their ecologies, and environments.

## Survey Methodology

So far there were no comprehensive reviews concerning the use of freshwater and marine sponge spicules. To provide an overview of the most relevant articles dealing with the application of sponge skeletal elements in taxonomic, ecological, and environmental studies I went through the records of Google Scholar and PubMed. I used the search terms “sponge spicules”, “glass ramp”, “spiculite”, and “spongillite” (see Table 1 for the numbers of obtained records). Note that the term “sponge spicules” returns a very high number of records in Google Scholar. The results were examined one-by-one and the vast majority of the records turned out to be irrelevant for the present paper. Therefore, additional search was conducted, this time limited only to works containing the searched term in the title. The online search was performed in August 2020. Expectedly, the records from both databases overlapped. Each record was further explored in detail and additional references were obtained from the literature cited in these works. The literature that was not available online—mostly printed-only manuscripts from the first half of the 20th century and old, 19th century classic paleontological works—were obtained through the Library of the Institute of Paleobiology, Polish Academy of Sciences, Warsaw, Poland. Following the survey, I compiled a database (Supplemental Information 1) comprising a list of the most relevant articles (including the year of publication, authorship, language of the publication, type of environment [marine/freshwater], type of studies (e.g., related to sponge taxonomy, water properties, climate etc.), geographic area of investigation, and the age of the investigated material). In the sections below, I discuss the papers that appear to be of special relevance for the present contribution. In these papers, the disassociated sponge spicules had to be (i) the only, (ii) the main, or (iii) a noteworthy component used for the taxonomic, ecological, and environmental studies. Despite that efforts were made to provide an exhaustive list of studies, it cannot be rules out that some relevant papers were unintentionally omitted, for example due to their absence in the explored search engines.

**Table 1:** The number of records obtained through Google Scholar and PubMed using the search terms “sponge spicules”, “glass ramp”, “spiculite” and “spongillite”.

Term	Sponge spicules		Glass ramp		Spiculite		Spongillite	
	Records	Relevant	Records	Relevant	Records	Relevant	Records	Relevant
Google Scholar	18,500 (184: in title)	28	150	12	1130	35	47	15
PubMed	367	6	58	0	1	0	0	0

### From formation to deposition: the ‘life cycle’ of spicules

**Figure 1:**
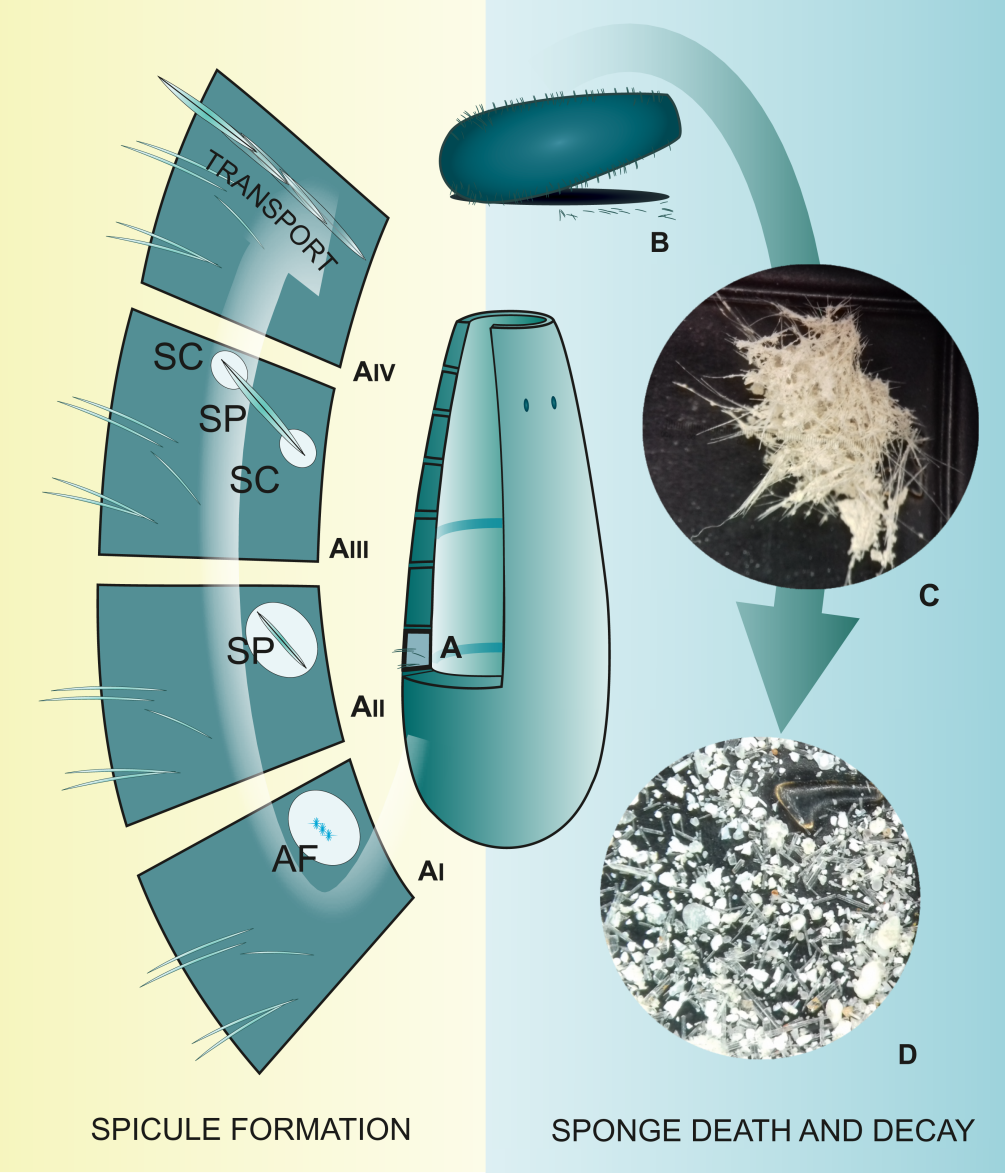
Spicule “life cycle”. (A) Spicule development in the mesohyl; (AI) Formation of spicule axial filament (AF); (AII) Spicule (SP) growth within the sclerocyte; (AIII) Spicule growth with two sclerocytes (SC) on spicule tips; (AIV) Transport of mature spicule within the sponge body; (B) Sponge death and body decay; (C) Detached sponge fragment with spicules; (D) Disassociated spicules. (C and D) Photo credit: Magdalena Łukowiak.

The formation of spicules is controlled genetically (Krasko et al., 2000). In most cases, the first growth phase is intracellular; it starts in sclerocytes (amoeboid cells responsible for spicule formation) in mesohyl (Custódio, Hadju & Muricy, 2002; Müller et al., 2005) and is mediated by silicatein, a special enzyme that initiates formation of the axial filament (harbored by the axial canal) which provides the vertical axis of the spicule (Shimizu et al., 1998; Fig. 1AI). The axial canal is filled with organic proteinaceous material which usually extends to the tip of the newly-formed spicule (Simpson, Langenbruch & Scalera-Liaci, 1985). The cross-section of the axial canal differs across major sponge clades that produce siliceous spicules (it is triangular in demosponges (Uriz, Turon & Becerro, 2000), irregular in homoscleromorphs (Uriz et al., 2003) and quadrangular in hexactinellids (Uriz, 2006)). In calcareans (producing calcareous spicules) the axial canal is not developed (Uriz, 2006). The geometry and the length of the axial filament determines the shape of the spicule (Pisera, 2003). In desmoid spicules of ‘lithistids’ (an informal group of demosponges with articulated skeletons), however, the axial filament is shorter than the spicule arms and it is possible that only organic molecules are involved in the spicule-forming process (Pisera, 2003).

During formation of the siliceous spicules (Calcarea displays different mechanisms of spicule biomineralization), sponges obtain silicon in the form of soluble silicic acid and deposit it around the axial filament (see Uriz, Turon & Becerro, 2000; Uriz et al., 2003), within a special membrane called silicalemma (Simpson, 1984, 1989). Silica is first laid out as small 2 µm granules (Uriz, Turon & Becerro, 2000; Pisera, 2003) that are fused to bigger spheres (or fused together within process of biosintering in Hexactinellida; Müller et al., 2009). After some time, amorphous silica is added, forming evenly-deposited concentric layers (Uriz et al., 2003; Fig. 1AII), separated from each other by ultrathin organic interlayers (Werner et al., 2015). At this stage, immature spicules are secreted from the sclerocyte and covered by pseudopodia of one to several cells (Fig. 1AIII); the process of silica deposition and spicule growth continues (Uriz, Turon & Becerro, 2000).

After completing the deposition of silica (or during this phase), the spicule is transported to the right place in the sponge body by crawling mesohyl cells, where spongocytes secrete spongin fibrils around them and connect them with adjacent spicules (Uriz et al., 2003; Fig. 1AIV). In some hexactinellids, that are characterized by rigid skeleton, the fusion of spicules appears to occur parallel to spicule secretion (Reiswig, 2002).

When sponges are alive, their spicules provide a structural “framework”. Following their death (Fig. 1B), the body and the skeleton structure, especially that of demosponges in which the spicules are connected to each other only by perishable collagen fibers (Fig. 1C), rapidly disintegrate leaving the spicules “free” (Fig. 1D); thus, sponges are rarely wholly preserved in the fossil record. Their spicules, however, are incorporated into sediments, often becoming one of the main components of sedimentary rocks (Rützler & Macintyre, 1978; Harrison & Warner, 1986). Sometimes spicules accumulate into enormous agglomerations called spicule mats or beds (Gutt, Böhmer & Dimmler, 2013). These accumulations are characteristic for polar waters (Koltun, 1968; Gutt, 2007). Spicules can fossilize to form special type of rocks called the spiculites (spongillites for freshwater sponge spicules); these types of rocks are known globally (Cayeux, 1929; Cavaroc & Ferm, 1968; Gammon, 1978; Reid et al., 2008; Schindler, Wuttke & Poschmann, 2008), and have been formed through the whole Phanerozoic (Gammon, 1978). Biosiliceous sedimentation occasionally results in the formation of spiculitic cherts (in so called glass ramps) which are recorded from the Permian to Eocene of many parts of the world (Gates, James & Beauchamp, 2004; Ritterbush, 2019).

### Assigning loose spicules to taxa

**Figure 2:**
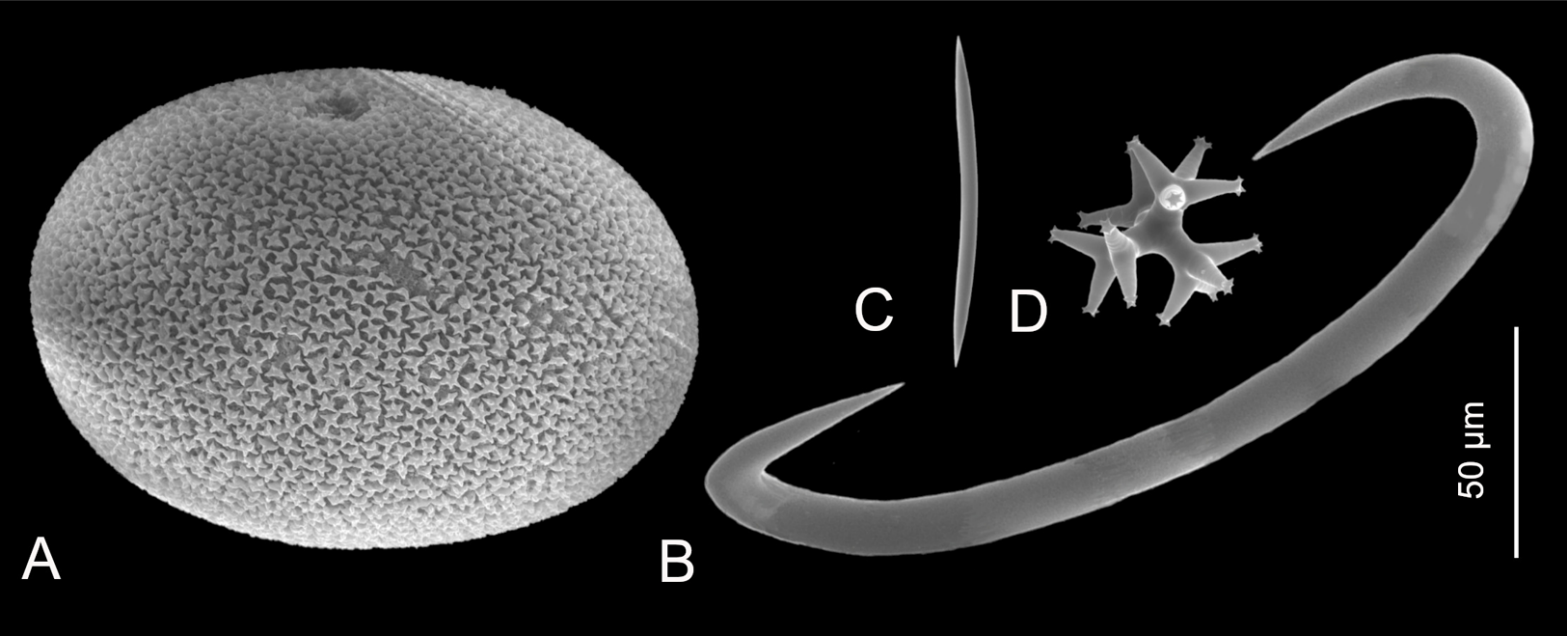
Sizes of different spicule types of marine sponges. (A) Microsclere (sterraster) of *Geodia* spp.; (B) Microsclere (sigma) of *Mycale* (*Mycale*) *quadripartita*; (C) Megasclere (oxea) of *Haliclona* (*Haliclona*) *epiphytica*; (D) Spicule tetralophose calthrop of homoscleromorph *Plakina* cf. *atka*. (A) Photo credit: Magdalena Łukowiak; (B and C) Photo credit: Rob van Soest; (D) Photo credit: Andrzej Pisera.

The use of sponge spicules in taxonomic, ecological, and environmental studies relies on the proper understanding of spicule morphologies and their distribution among sponge taxa. Owing to the great variety of spicule morphotypes, spicules are conventionally divided based on their size into micro- and megascleres. The microscleres typically cover spicules that are up to 150 µm long. The megascleres, in turn, comprise larger spicules and their usual size is up to a few millimeters; though in some extreme cases, as in the basal spicule of the hexactinellid *Monorhaphis chuni*, that is the largest known biosilica structure, they reach 3 m in length (Lévi, 1973, 1989; Wang et al., 2007). The division into micro- and megascleres, however, is not strict and the boundary between these two categories can be somewhat blurry. Some microscleres, such as the sterrasters of *Geodia* spp. or sigmas of *Mycale* spp. (Figs. 2A and 2B, respectively), can reach the size or even become larger than an average megasclere. In turn, some megascleres, such as the oxeas of *Haliclona* (*Haliclona*) *epiphytica* are small and do not exceed 100 µm (Fig. 2C; Uriz et al., 2003). The spicules in some homoscleromorph sponges, such as some plakinids (e.g., *Plakina* cf. *atka*; Uriz et al., 2003), are of ‘intermediate’ size (see Fig. 2D). The same applies for ‘mesoscleres’, the spicules that develop in hexactinellids (Reiswig, 2002).

**Figure 3:**
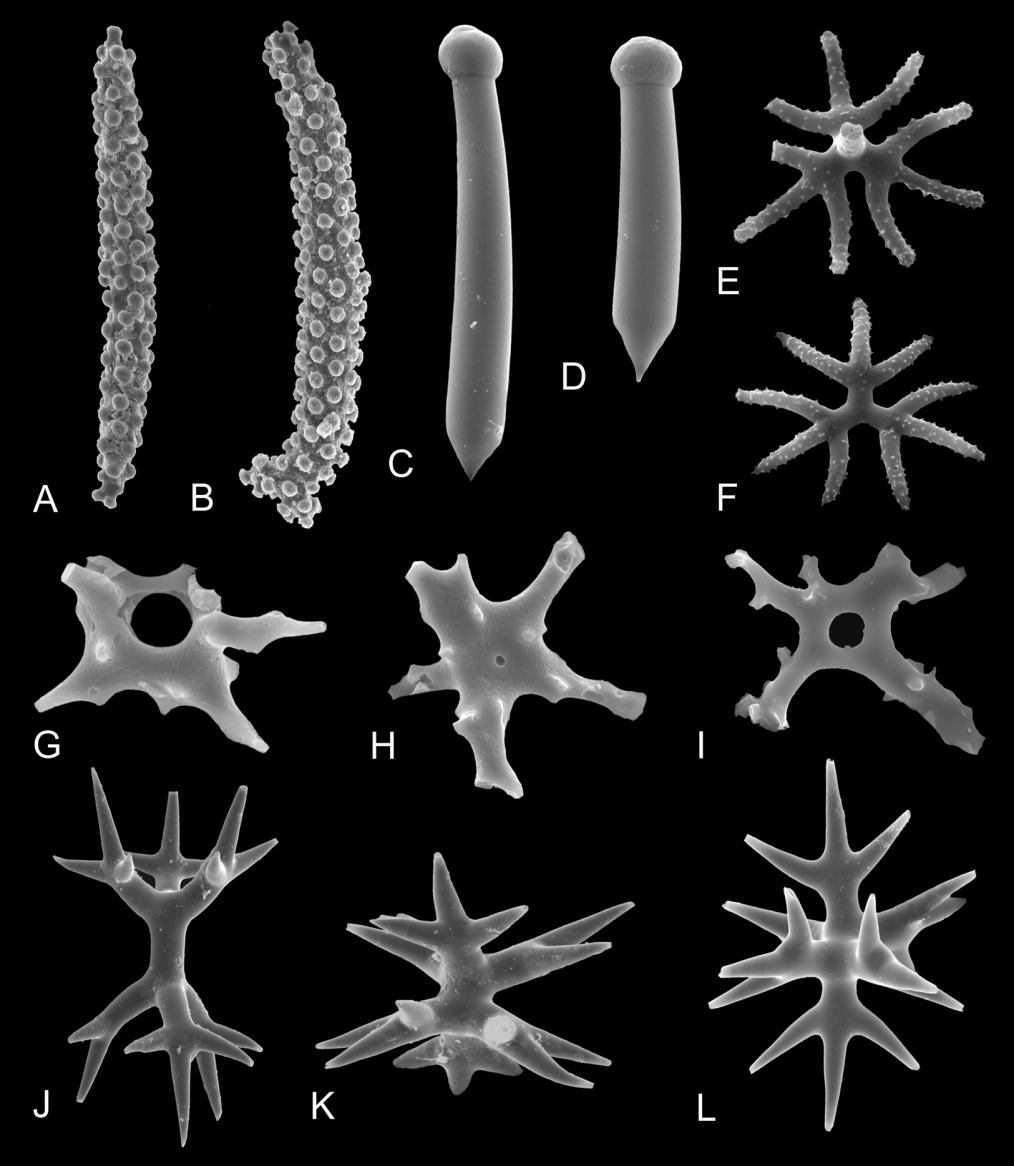
Morphological diversity of selected marine sponge spicules. (A and B) Tuberculate diactines of *Alectona wallichii*; (C and D) Subtylostyles of *Cliona mucronata*; (E and F) Shortshafted triaenes of *Thrombus abyssi*; (G–I) Desmas of *Vetulina* sp.; (J–L) Amphitriaenes of *Samus anonymus*. (A–F and J–L) Photo credit: Magdalena Łukowiak; (G–I) Photo credit: Andrzej Pisera. Not to scale.

**Figure 4:**
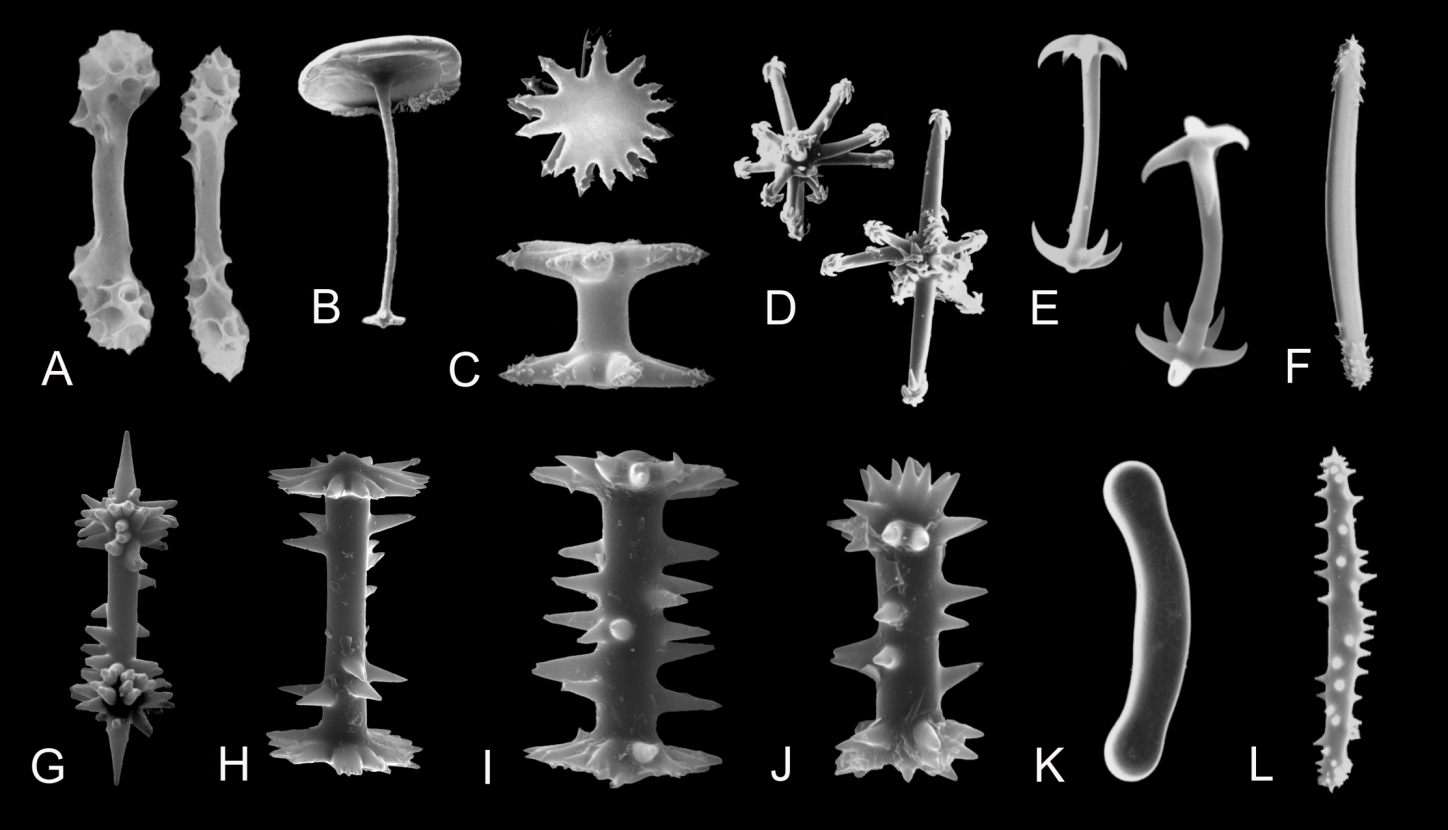
Morphology of gemmuloscleres and spicules of freshwater sponges of the order Spongillida. (A) Gemmuloscleres of *Anheteromeyenia argyrosperma*; (B) Tubelliform gemmulosclere of *Acalle recurvata*; (C) Birotules of *Ephydatia*; (D) Aster-like microscleres of *Dosilia plumosa*; (E) Pseudomicrobirotules of *Corvospongilla burmanica*; (F) Strongyloxeas of *Baikalospongia bacillifera*; (G–J) Birotules (gemmuloscleres) of *Ephydatia* cf. *facunda*; (K) Strongyle of *Potamolepis marshalli*; (L) Microxea of *Drulia browni*. (A–L) Photo credit: Andrzej Pisera; Not to scale.

Despite that there is only just over 12 basic types of megascleres and 25 types of microscleres recognized in demosponges, and 20 basic types of megascleres and 24 types of microscleres in hexactinellids (Boury-Esnault & Rützler, 1997; Tabachnick & Reiswig, 2002), one type of spicules in homoscleromorphs (Muricy & Díaz, 2002), and three basic types in calcareans (Manuel et al., 2002), actual variation of spicule morphologies makes the real number of different morphotypes counted in hundreds (e.g., Figs. 3A–3L and 4A–4L; e.g., Łukowiak, 2015).

However, despite the high variation, assignments of particular spicule morphotypes to taxa remain complicated as the relation between the types of spicules and sponge taxa is rarely straightforward. Even though some spicule types are unique to species—e.g., the diactines of *Alectona wallichii* (Figs. 3A and 3B), the subtylostyles of *Cliona mucronata* (Figs. 3C and 3D), the short acanthotriaenes of *Thrombus* spp. (Figs. 3E and 3F), the desmas of *Vetulina* spp. (Figs. 3G–3I), and the amphitriaenes of *Samus anonymus* (Figs. 3J–3L)—other spicules (e.g., oxeas) are widespread and develop in numerous sponge clades in which many of them originated independently (Cárdenas et al., 2011).

**Figure 5:**
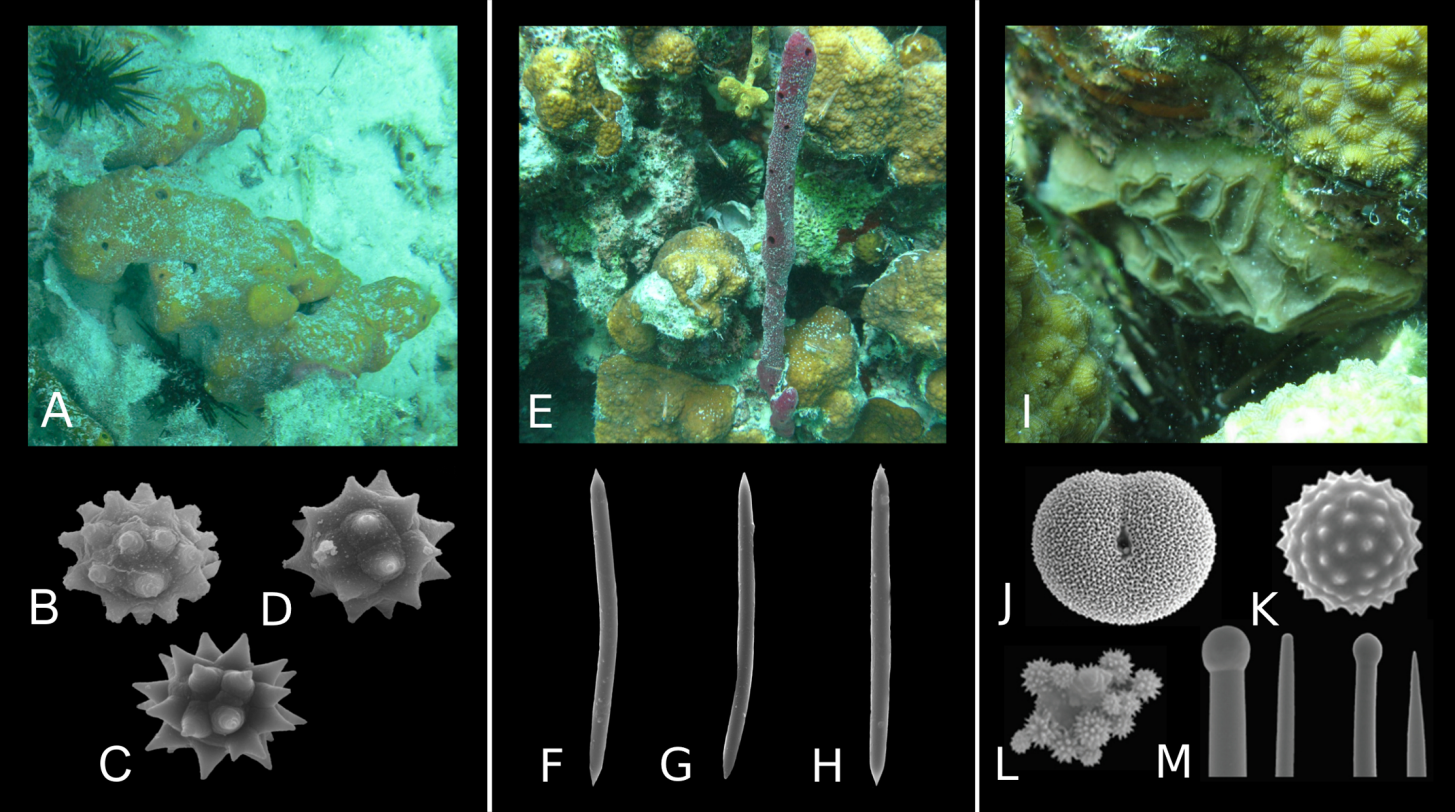
Diversity of spicule morphology and sets of selected sponge species. (A) Sponge species with one type of highly diagnostic spicules: *Chondrilla caribensis* (forma *caribensis*); (B–D) Spherasters; (E) Sponge species with one type of morphologically simple, non-diagnostic spicules: *Amphimedon compressa*; (F–H) Oxeas; (I) Sponge species with a set of diagnostic spicule types: *Placospongia melobesioides*; (J) Selenaster; (K) Spheraster; (L) Spherules; (M) Tylostyles of two size categories. (A–I) Photo credit: Magdalena Łukowiak; (J–M). Becking (2013) (published under the license CC BY 3.0). Not to scale.

Even though the formation of spicules is controlled genetically, silica saturation also plays a role in spicule development. In some unfavorable conditions (undersaturated water), some types of microscleres are not produced (Uriz & Maldonado, 1995; Maldonado et al., 1999) which results in a reduced set of spicules. Variation in spicule distribution can be further observed at the individual scale. Many sponge species produce skeletons that comprise a single spicule morphotype, such as *Chondrilla caribensis* and *Amphimedon compressa*. However, while the spherasters of *Ch. caribensis* are easily recognizable (Figs. 5A–5D), the oxeas of *A. compressa* cannot be unambiguously referred to the taxon (Figs. 5E–5H). Other sponge taxa, in turn, comprise several spicule morphotypes (e.g., *Placospongia melobesioides*; Figs. 5I–5M). The morphotypes of spicules and their quantity can vary greatly within species as well (Hartman, 1981).

### Review of the utility of loose spicules in neontological and paleontological disciplines

Inoue (1984, 1985), Bertolino et al. (2012, 2017a, 2017b, 2019) and Łukowiak, Pisera & O’Dea (2013) investigated the relation between living sponges and spicule assemblages. The studies concerned marine sediment spicules, their preservation potential and how faithfully they reflect the living sponge communities. Both, surface sediment spicules (Łukowiak, Pisera & O’Dea, 2013) and spicules from assemblages of coralligenous conglomerates (Bertolino et al., 2014, 2017a, 2017b, 2019) were shown to reflect the living sponge communities to a good degree of accuracy and can be used, with some cautions, for reconstructing former sponge communities. In turn, Matteuzzo et al. (2015) investigated the factors that influence the formation of spicules in freshwater sponges. They showed that the sequential production of a complex set of spicules (alpha megascleres followed by microscleres, gemmuloscleres and beta megascleres) in the neotropical freshwater sponge *Metania spinata* depends on the water level, temperature, and silicon concentration. They proved that the environmental reconstructions based on the presence or absence of alpha megascleres and gemmuloscleres of freshwater sponges in sediments can estimate the values of these environmental factors.

### Sponge spicules in taxonomic studies

#### Reconstructing sponge communities

Numerous studies have used assemblages of disassociated spicules for reconstructions of former sponge associations. The first attempts to reconstruct sponge communities based on loose spicules at a larger scale were published at the turn of the 19th and 20th century for example in the works of Carter (1871) and Dunikowski (1882). In turn, the studies of Hinde and Holmes (Hinde & Holmes, 1892; Hinde, 1910) focused on rich spicule assemblages of the Southern Hemisphere and revealed an apparent richness of the Eocene sponge fauna of New Zealand and Western Australia, respectively. Ever since these pivotal attempts to comprehend the complexity of sponge spicule associations, fossil, sub-fossil, and recent spicules have become the subject of numerous locality- or region-specific studies that focused on assessing the sponge community compositions (Schrammen, 1924; Reif, 1967; Moczydłowska & Paruch-Kulczycka, 1978; Mostler, 1976, 1990; Hartman, Wendt & Wiedenmayer, 1980; Gruber & Reitner, 1991; Kaesler, 2004 and the literature cited therein). Wiedenmayer (1994) summarized the knowledge on the post-Paleozoic sponge spicule record from different stratigraphic units and compared the spicules derived from the fossil record with their possible counterparts present in modern sponge taxa. The results of these investigations led him to provide discussion of the origins and history of some sponge groups, including their ecological dependencies and paleobiogeography. The studies of sponge spicules further intensified in subsequent years (Brasier, Green & Shields, 1997; Castellani et al., 2012; Carrera & Maletz, 2014; Łukowiak, Pisera & Stefanska, 2019). For instance, Łukowiak (2015) supplemented the work of Hinde (1910) and revealed the richness of the sponge community of southern coasts of the Australian continent. The reconstructed community was later compared with that of New Zealand (Łukowiak, 2016a) that was initially described by Hinde & Holmes (1892). Beresi (2003) reviewed the studies on Cambrian sponge spicules (and chancelloriid sclerites) from the Argentine Precordillera, while Frisone et al. (2014) described isolated spicules form the Eocene of Italy. Interestingly, despite that there are many complications associated with studies of fossil loose sponge spicules, analyses of the spicular record from modern surficial sediments may be helpful in traditional faunistic studies. They can detect the presence of extant sponges that can be easily overlooked due to their small size, or cryptic or excavating nature. For instance, Łukowiak (2016b) focused on the sponge spicules from the lagoon reef of Bocas del Toro, Panama, and noticed the presence of highly diagnostic spicules belonging to cryptic and excavating sponges that had not been noted from that area before. Similar studies can be performed in freshwater habitats to consider the history the sponge communities (Wilkins et al., 1991) or of individual sponge taxa (Hall & Herrmann, 1980).

#### Assessing sponge community dynamics in time

The information obtained from loose spicules has been also applied to investigate the changes in the record of spicule assemblages through time. Such studies require spicule-rich sediment portions (Volkmer-Ribeiro, De Ezcurra & Parolin, 2007) and the best results are obtained when an uninterrupted spicule record is available. It seems that absolutely-dated sediment cores are the best way to obtain continuous record of the changes in spicule assemblages (Bertolino et al., 2014, 2017a, 2017b, 2019; Gaino et al., 2012; Łukowiak et al., 2018; Rasbold et al., 2019a).

The spicules obtained from different time intervals can be further processed by means of quantitative (Volkmer-Ribeiro et al., 2006; Bertolino et al., 2014; Łukowiak et al., 2018; Rasbold et al., 2019a), semiquantitative (Karabanov et al., 2000), and qualitative (Yang, Duthie & Delorme, 1993; Bertolino et al., 2014) methods. Such approaches allow to explore the stability (Bertolino et al., 2014) and dynamics (Bertolino et al., 2017b; Łukowiak et al., 2018) of the sponge associations in a given place over the millennial time span. They also allow to compare the recent and ancient sponge communities (Łukowiak, 2016a; Łukowiak et al., 2018; Bertolino et al., 2014, 2017a, 2017b, 2019). Bertolino et al. (2017b) investigated fluctuations in the sponge community structure from the coralligenous biogenic build-ups from the Liguarian Sea (Western Mediterranean). They compared sponge faunas from the last 3,500 years (and from 40 YBP in particular) with the modern sponge community of this place, showing turning points in sponge diversity and abundance. They inferred a considerable reduction of species richness that occurred during a time interval of about 40 years (1973–2014) and that affected mainly the massive/erect sponges; particularly those of the clade Keratosa and, to a lesser degree, encrusting and cavity dwelling sponges (Bertolino et al., 2017b).

In turn, Łukowiak et al. (2018) confronted the modern sponge community of Bocas del Toro, Caribbean Panama, with the spicule record from the same area covering the last 900 years of sponge community dynamics. In their study, Łukowiak et al. (2018) recognized changes in the structure of the sponge community (expressed through the decrease in the number of spherical and ovoid spicules belonging mostly to some chondrillids, *Placospongia* spp. and *Geodia* spp., and the increase in the numbers of monaxonic spicules, mostly of haplosclerids and axinellids) and investigated the correlation between their data with records of contemporaneous reef inhabitants. As main sponge predators in this area are turtles and fishes (parrot- and anglerfishes), and the turtles feed mostly on sponges with spherical spicules, they concluded that the increase in the numbers of spherical spicules in the sediment relative to monaxons could be connected with the overfishing of the hawksbill turtle *Eretmochelys imbricata* that became gradually less abundant at that time.

### Sponge spicules as proxies for reconstructions of ecological dependencies, environmental conditions, and paleoclimate

#### Ecological and environmental reconstructions

**Figure 6:**
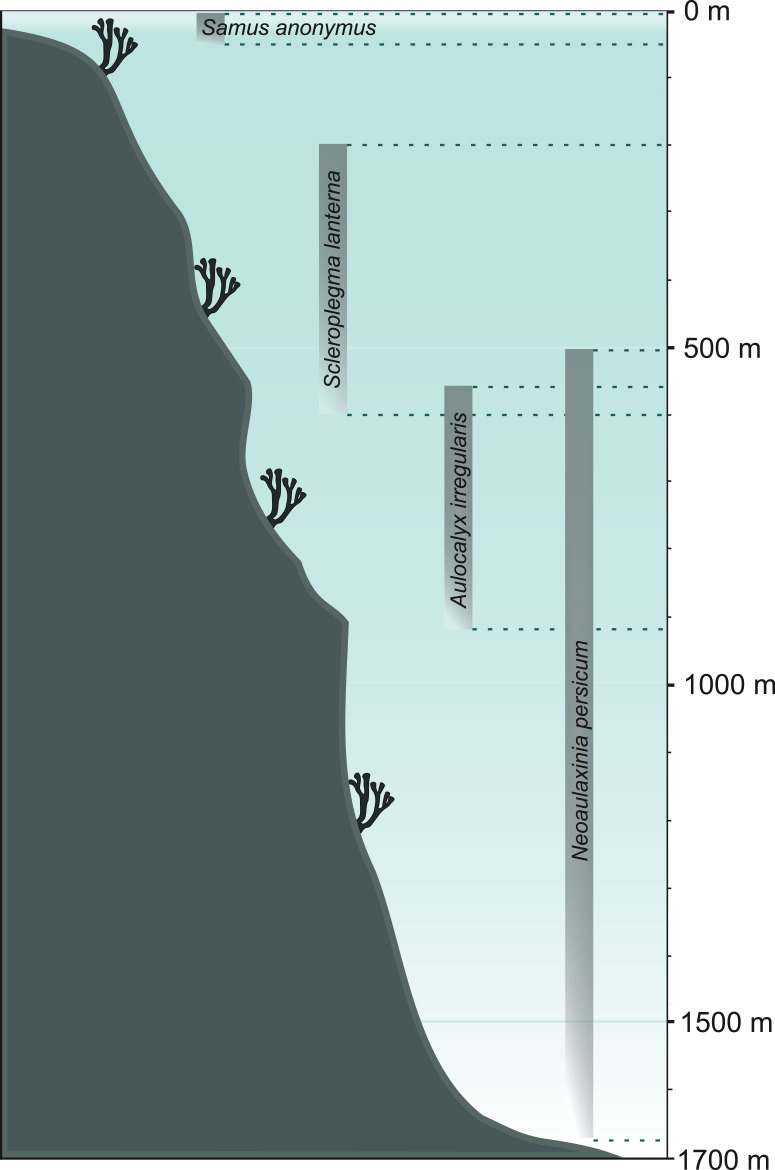
Bathymetrical range of selected sponge species. Demosponge *Samus anonymus* (up to 50 m), hexactinellid *Scleroplegma lanterna* (~100–600 m), hexactinellid *Aulocalyx irregularis* (~550–915 m), lithistid demosponge *Neoaulaxinia persicum* (~500–1,700 m).

Recognition of the characteristics of former sponge communities can initiate larger-scale reconstructions of the environmental conditions in the studied area. Once the loose spicules are assigned to their respective taxa, the next crucial step is to become familiar with their ecology (Volkmer-Ribeiro & Machado, 2007); that is, habitat requirements and environmental preferences of particular sponge species or larger clades (see Supplemental Information 2). For example, Koltun (1960) already pointed out the existence of sponge taxa of specific environmental requirements which make them potential indicators of water salinity, temperature, or depth. Recognized sponge communities can provide information about the water regime, flow, and velocity (lentic vs. lotic conditions; for example, Parolin, Volkmer-Ribeiro & Stevaux, 2007, 2008; Machado, Volkmer-Ribeiro & Iannuzzi, 2012; Kuerten et al., 2013), pH (Harrison & Warner, 1986; Pisera & Saez, 2003), light intensity (e.g., Harrison, 1974; Supplemental Information 2), temperature (e.g., Gammon, James & Pisera, 2000; Gaino et al., 2012), currents (e.g., Molina-Cruz, 1991), salinity (e.g., Cumming, Wilson & Smol, 1993), and depth (e.g., Hinde & Holmes, 1892; Pisera, Cachao & Da Silva, 2006; Łukowiak, Pisera & Schlögl, 2014; Łukowiak, 2016a; see Fig. 6). Also, less obvious parameters can be reconstructed through the investigations that use sediment spicules, such as dissolved silica concentrations (Kratz et al., 1991). In their work, Kratz et al. (1991) measured the width of megascleres obtained from lake deposits to estimate the silica concentration in the last 12,000 years in the area of Wisconsin (USA). They concluded that the concentrations of dissolved silica were gradually decreasing over that time. However, Kratz et al. (1991) did not provide precise assignment of the spicules to any of the three possible species (*Spongilla lacustris*, *Corvomeyania everetti*, and *Eunapius fragilis*) rising concerns that the size shift may have actually resulted from species replacement. A possible differential dissolution of spicules as a function of depth could also influence their size. It is essential to note, however, that the authors were aware of these problems.

Sponge spicules are also common components of soils (Kaczorek et al., 2018 and the literature cited therein) and can provide information about soil origins (Jones & Beavers, 1963; Wilding & Drees, 1968; Harrison, 1988b; Schwandes & Collins, 1994). In most cases, the spicules found in soils belong to freshwater sponges (Harrison, 1988b) and are indicative of the fluvial origin of the deposits (Chauvel, Walker & Lucas, 1996). The distribution, type and degree of fracturing of spicules in soil can help to reveal whether the sediments are autochthonous or allochthonous, and, if the latter, where they originated (Wilding & Drees, 1968; Schwandes & Collins, 1994). Schwandes & Collins (1994) showed that in well-drained soils the number of spicules is smaller than in those that are poorly-drained and noticed that in the dry pounds the number of spicules increased towards the pond center, thus clarifying the precise location of these ancient waterbodies.

Sponge spicules are also found in other types of wet terrestrial environments; in waterlogged soils, hydric soils (Schwandes & Collins, 1994), and marshland (Volkmer-Ribeiro, 1992). Apart from providing information on the origins of soils, spicules can reveal the occurrence, duration, and intensity of floods (Golyeva, 2008) or the presence of eolian transport (Wilding & Drees, 1968).

Through the use of demosponge spicules and by applying the facies analysis it is possible to reconstruct sedimentary environment of spicule-rich deposits (Mehl & Lehnert, 1997; Frisone et al., 2014). The recognition of sponge taxa with very narrow and specific environmental preferences can facilitate the reconstructions of water depth (Fig. 6), temperature, and climate (Parolin, Volkmer-Ribeiro & Stevaux, 2008; Pisera, Siver & Wolfe, 2016), though it is necessary to bear in mind that some sponges (notably hexactinellids and lithistids) that are generally thought to be deep-water indicators, are also reported form shallow waters (2 m; Leys et al., 2004; Perez et al., 2004). Therefore, bathymetric reconstructions should not be based on a single taxon.

However, such actualistic approach needs to be applied with caution. Under certain favorable conditions (e.g., calm hydrodynamic setting and high water dissolved silica (DSi) concentrations (Alvarez et al., 2017)), modern sponges that are interpreted as indicators of cold deep waters may have inhabited warm and shallow settings in the past (Gammon, James & Pisera, 2000).

Sponge distribution is closely associated with water nutrient level, including water DSi concentrations (for more details see Alvarez et al., 2017). Sponge assemblages were studied to estimate the distributions of sponges along a DSi gradient and to assess the validity of fossil sponges as a paleoecological tool for inferring DSi concentrations in the past oceans (Alvarez et al., 2017). The study showed a correlation between the presence of hexactinellid sponges in an environment and high DSi levels; for the other sponge groups the linkage was not so straightforward and depth could have been another factor that could have some impact on the spatial distribution of sponge assemblages (Alvarez et al., 2017).

The reconstructions of geographical ranges of sponge taxa, in turn, lead to new paleobiogeographical and ecological inferences. A southern Australian shallow water sponge community reconstructed by Łukowiak (2016a) broadened the geographical ranges of some sponge taxa (e.g., *Dotona pulchella* and *Mycale* (*Rhaphidotheca*) *loricata*) in the late Eocene. Their bathymetrical range changed too as today they occupy deep water niches and are considered to be Tethyan relicts. The broader geographical occurrences in the geological past further explain the disconnected distribution of some sponge groups in modern times and possibly elucidate their migration pathways. When compared the fossil sponge community of southern Australia with the recent one, the taxonomic composition of the sponge fauna in this region appears to be relatively stable since the Eocene (Łukowiak, 2016a).

Of special interest and importance are sponges inhabiting freshwater environments because they represent accurate environmental indicators (Harrison, 1988a, 1988b; Volkmer-Ribeiro & Turcq, 1996; Frost, 2001; Volkmer-Ribeiro, De Ezcurra & Parolin, 2007; Parolin, Volkmer-Ribeiro & Stevaux, 2008). Their spicules, together with gemmuloscleres (the spicules of sponge resting bodies) (Manconi & Pronzato, 2002), are widely used as a proxy for reconstructions of the parameters associated with the water quality (Yang, Duthie & Delorme, 1993).

Some characteristics of sponge spicules can change depending on environmental parameters, such as the silica concentrations of the habitat (Turner, 1985; Matteuzzo et al., 2015), which may provide a paleolimnological measure of long-term silica dynamics or past water-chemistry conditions (Kratz et al., 1991). Such inferences became possible mainly owing to the studies of the environmental preferences of modern freshwater sponges (Harrison, 1974, 1977, 1979; Harrison & Harrison, 1977, 1979; Harrison, Gleason & Stone, 1979; Poirrier, 1969, 1974; Volkmer-Ribeiro, 1981; Evans & Montagnes, 2019). The development in our understanding of the base-line ecological parameters of many extant freshwater sponge species was gathered and summarized by Harrison (1988b) and the literature cited therein who provided a comprehensive and useful key to environmental preferences of North American freshwater sponge species that could be applied in paleolimnological studies (see Supplemental Information 2; for example, Racek, 1966, 1970, 1974; Harrison, 1990).

Importantly, the spicular data can be used for quantitative studies irrespective of the species assignment. The abundance of sponge spicules in combination with the data obtained from other organisms (e.g., diatoms, chrysophyte cysts, plant phytoliths, and scales) is an indicator of suitable habitat conditions and the decrease in the numbers of spicules can be interpreted to be due to the deterioration of water quality (Yang, Duthie & Delorme, 1993) or changes of past salinity conditions (Cumming, Wilson & Smol, 1993). Gaiser et al. (2004) reconstructed a complex 5,500-year-long hydrological history of a temporary pond in the Savannah River Site, South Carolina (USA). They reconstructed a transformation from a vegetated marsh to an open, permanently flooded water body about 3,750–4,630 YBP. The return to a wetland community took place ~3,750 YBP (Gaiser et al., 2004). Even though the authors recognized the presence of three sponge species in their assemblage, they did not explore whether their occurrence is indicative of an important environmental information (water pH, conductivity). The most comprehensive assessment of the use of freshwater sponge spicules for paleoenvironmental reconstructions was conducted in riverine and lentic systems in Caatinga (Santos et al., 2016) and Pantanal wetlands ecosystems (McGlue et al., 2012; Kuerten et al., 2013; Rasbold et al., 2019b) in Brazil. Parolin, Volkmer-Ribeiro & Stevaux (2007, 2008), Guerreiro et al. (2013) and Zviejkovski et al. (2017) focused mostly on reconstructing the history of lake sediments on colluvial-alluvial terraces in the Upper Paraná River, while Rasbold et al. (2019a) investigated the history of island deposits. Rasbold et al. (2019a) reconstructed the evolution and origin of two closely located riverine islands (Bandeirantes and Grande) during the late Pleistocene to Holocene in the northern Brazil (Upper Paraná River). The spicules of freshwater sponges were used to reconstruct the assemblage composition, the relationship between species richness and frequency, and spicule preservation, as well as to complement the facies analysis. Rasbold et al. (2019a) revealed the differences in formation of these two islands. For example, in one of them the lacustrine environments were predominant since the mid-Holocene (the lentic environment is inferred from the presence of spicules of *Tubella variabilis* and *Radiospongilla amazonensis*), whereas the second island was cut off from the river floodplain by channel avulsion and influenced by the river flow (as indicated by the presence of spicules of riverine species, including *Uruguaya corallioides*, *Oncosclera schubarti*, *O. navicela* and *Corvospongilla seckti*). In the coastal area of Rio Grande do Sul (S Brazil), in turn, the numbers and the morphotypes of sponge spicules (and gemmuloscleres) of several freshwater bodies provided evidence for a regular succession of seasonally drier periods (Volkmer-Ribeiro et al., 2006). Further, the study showed that the analysis of sediment spicules could also predict the evolution of this limnic area (Volkmer-Ribeiro et al., 2006). In the northern part of Brazil sponge spicules were used to investigate the origins of deposits of karstic lake (Machado, Volkmer-Ribeiro & Iannuzzi, 2016), while in central-west Brazil the evolution of the limnic system was reconstructed (Machado, Volkmer-Ribeiro & Iannuzzi, 2014). Machado, Volkmer-Ribeiro & Iannuzzi (2014) investigated sediments from about 52,000 to 27,500 YBP and recognized three stages of the development of the lake: the installation, establishment, and development stage. Following the recognition of environmental preferences of the identified sponge species, they estimated the weather patterns. For instance, the presence of spicules belonging to *Corvoheteromeyenia australis* indicated polar incursions. The presence of spicules of *Corvomeyenia thumi*, in turn, suggested that drier and hotter weather conditions might have been predominant for short time periods (between ~48,333 and 34,700 YBP). The history of the paleolake ended with complete filling of the basin and disappearance of the sponge spicules and can be correlated with the Last Glacial Maximum, which occurred between 25,000 and 11,000 YBP (Machado, Volkmer-Ribeiro & Iannuzzi, 2014).

#### Isotopes in sponge spicules as a geochemical proxy

The structural and chemical properties of siliceous shells of seawater diatoms were found to be archives of silicon isotope composition (δ^30^Si) and a proxy for past silicic acid utilization, thus providing insights into silicon cycling in ancient oceans (De La Rocha, Brzezinski & De Niro, 1997; De La Rocha et al., 1998; Racki & Cordey, 2000; Egan et al., 2012). These studies initiated the investigations of the use of silicon isotope composition in other marine organisms producing siliceous skeleton, including sponges (Douthitt, 1982; De La Rocha, 2003). Sponges, as bottom dwellers, provide information about deep-water dissolved silica (DSi) concentrations. The studies of Hendry & Robinson (2012) were conducted on materials picked from core-top sediments that were obtained from several different ocean basins. The spicules were evaluated with respect to whether the Si isotopic fractionation depends on the geographic distribution, salinity, and water temperature. It was discovered that the relationship between Si(OH)_4_ and δ^30^Si in sponge spicules is the same in different oceans and does not depend on water temperature and salinity. Thus, global ocean silicon cycling can be inferred through quantification of the changes in deep water dissolved silicon concentrations (Fontorbe et al., 2017; Hendry et al., 2019). The silicon isotopic fractionation appears to be biologically controlled due to preferential uptake of the light isotope (Wille et al. 2010) and is a function of silicic acid concentration (Hendry & Robinson, 2012). Also, Hendry et al. (2010a) showed that there are no significant post-depositional effects or early diagenetic overprints on Si fractionation in deep sea sponge spicules. It was further showed that the relationship between δ^29^Si and δ^30^Si in sponges is consistent with kinetic fractionation during biomineralization and that fossil spicules preserve the primary δ^30^Si signal recorded in living sponges (Hendry et al., 2010a). However, Cassarino et al. (2018) revealed the differences in silicification mechanism between the two major clades, demosponges and hexactinellids. The fused dictyonal frameworks of hexactinellids, characterized by secondary silicification, exhibit extremely light δ^30^Si signatures. This is probably due to the enzymes that mediate silica deposition. The differences in spicule structure, that is, the level of fusion of the skeleton and hence, the presence of the secondary silica deposition as well as the presence of considerable amounts of organic molecules, may also play an important role in silica fractionation (Cassarino et al., 2018). Thus, sponges with the dictyonal framework do not fit the asymptotic relationship with DSi. Also, specific groups of sponges (carnivorous or those with hypersilicified or giant spicules) give anomalous geochemical signatures (Hendry et al., 2019). Hendry et al. (2019) further revealed differences in isotopic fractionation between mixed-species and monospecific sponge assemblages, showing a smaller variability among monospecific aggregations (Hendry et al., 2019). Thus, the reconstructions of past water silica should be made on sponges with certain spicule types; that is, using the spicules of demosponges and hexactinellids, except those whose skeletons show the dictyonal framework and those with abnormal morphologies (Hendry et al., 2019).

Nevertheless, sponge spicules seem to be among the most promising sources for the reconstruction of deep water silicic acid concentration over the geological time. Deep sea sponge spicules and diatoms from sediment cores obtained in the Scotia Sea (Southern Ocean) have shown that water Si(OH)_4_ concentrations can be reconstructed for sediments deposited within the last tens of thousands of years (Hendry et al., 2010b); though, the reconstructions of water dissolved silicon level have been also inferred from spicules originating from the Eocene (De La Rocha, 2003; Fontorbe et al., 2016, 2017). Fontorbe et al. (2017) used the silicon isotopic composition of sponge spicules and radiolarian tests in 50–23 Ma timespan (Eocene and Oligocene) in order to reconstruct deep-water silica levels and to examine upper ocean δ^30^Si. The decrease of δ^30^Si and hence the higher dissolved Si concentrations in sponge spicules was interpreted as being related to the shift towards a solely Southern Ocean source of deep-water in the Pacific during the Paleogene (Fontorbe et al., 2017). Although the studies aiming to recreate the whole-ocean silica cycling have not been conducted on materials older than Eocene (De La Rocha, 2003; Fontorbe et al., 2017), they do not seem to be limited by time; rather, they depend on the quality of the spicular record.

Other studies applied germanium to silicon ratios (Ge/Si) from the siliceous sponge spicules to trace the Si sources and cycling (indicators, among others, of continental weathering and hydrothermal activity). Sutton (2011) showed that the reconstruction of Si concentrations in ancient water is more accurate when comparing two geochemical proxies for Si utilization, Ge/Si and Si isotope composition of siliceous organisms. She also suggested that the reconstruction of deep-water Si level from sponge spicules is more helpful in investigations of whole ocean changes than from the surface water provided by the Ge/Si or Si isotope composition of diatoms (Sutton, 2011). Ellwood et al. (2006) investigated the reconstructions of paleo-Ge and -Si concentrations using two models that depend on two scenarios of Ge incorporation into sponge silica. They explored which of them works best for reconstructions of paleo-Ge and which for paleo-Si concentrations (Ellwood et al., 2006).

Jochum et al. (2017) used the slowly-growing basal spicule (basalium) of the modern deep-sea sponge *Monorhaphis chuni* to reconstruct silica and Ge/Si ratios during the last deglacial period. The basalium of *M. chuni* was also used for the assessment of seawater paleotemperatures of East China Sea throughout the last 11,000 years (±3,000 years) by measuring isotopic composition and Mg/Ca ratios taken according to spicule growth, incrementally from the center of the spicule to its surface (Jochum et al., 2012).

### Pertinent gaps and new research directions

Despite that disassociated sponge spicules have already proven to be of great importance for taxonomic, paleoecological, and paleoenvironmental studies, a significant amount of work remains to be done to make a full use of their potential. The first concerns are associated with the “spicular analysis” (Harrison, Gleason & Stone, 1979), the assessment of loose sponge spicules and their assignment to sponge taxa. The method is still somewhat vaguely defined, largely “qualitative”, and relies on one’s expertise (the knowledge of spicule morphologies, distribution, and variation within and among sponge taxa); though larger-scale “spicular analyses” occasionally involve quantitative methods (Bertolino et al., 2014). In the case of morphologically characteristic spicules that appear in a limited number of taxa or are unique to a single species, the taxonomic assignment may be easy and the newly-obtained information unambiguous (Łukowiak, 2016b). In most cases, however, spicule assessments are challenging and the “spicular analysis” leads to several possible explanations for the observed spicule composition, especially when dealing with environments that show evidence for selective preservation (or removal of some components) or bear traces of transport over some distance. The processes acting on dead sponge bodies and their parts, involving the spicule transport, removal, and/or dissolution (especially of microsclere spicules; see Land, 1976; Rützler & Macintyre, 1978; Maldonado et al., 2005), are in general understudied. Still, the most significant gaps are associated with the investigation of the processes that take place when sponge body (or its part) decays. There are currently no data concerning the decomposition rate, nor the factors that can have an impact on this rate. Therefore, the understanding of the step-by-step disintegration of sponge individuals and the response of particular spicule morphotypes (such as microscleres) to removal pressures would significantly enhance the utility of spicule assemblages in paleoecological and paleoenvironmental investigations. Finally, due to lack of quantitative studies of ratios of particular spicule types in sponge bodies, the loose, disassociated skeletal elements (often comprising common spicule types that are present in numerous sponge taxa) in surface sediment usually represent an unknown number of sponge species of an unknown biomass. A quantitative model that would facilitate spicule assessments and sponge identification could be applied to provide a more precise reconstruction of the sedimentary environments without the need to “manually” assign each spicule type to sponge taxa. Even though spicular data have occasionally been assessed through quantitative methods (Bertolino et al., 2014; Rasbold et al., 2019a), such approach to the study of sponge spicules is still rarely used, despite that the usually high numbers of spicules, their widespread distribution, and differences in their morphologies appear to offer an excellent opportunity to conduct various multivariate and occurrence analyses.

The studies of isotopes in sponge spicules are of great importance and potential, either, as they provided an entirely new tool for reconstructions of silicic acid concentrations in the geological history (De La Rocha, 2003; Fontorbe et al., 2016). Nevertheless, substantial gaps still persist with respect to the knowledge of isotope fractionation processes. Aside from anomalous geochemical signatures in spicules of carnivorous sponges and those with hypersilicified skeletons or in giant spicules (Hendry et al., 2019), and in mixed sponge aggregations (Hendry et al., 2019), it remains to explore whether the silicon isotopic fractionation is dependent on the taxonomic position of the sponge and if it is constant during the spicule growth. The reliability of the results obtained from fossil spicules, however, is tightly coupled with the quality of the material (e.g., the degree and type of diagenetic alteration and dissolution processes).

Even though sponge spicules are well known for their significance as bearers of taxonomic, ecological, and environmental data, and have been investigated with that respect in numerous studies, their potential has yet to be fully exploited.

## Supplemental Information

10.7717/peerj.10601/supp-1 Supplemental Information 1 The list of the most relevant articles dealing with the application of sponge skeletal elements (spicules) in taxonomic, ecological, and environmental studies.

10.7717/peerj.10601/supp-2 Supplemental Information 2 Environmental preferences of selected freshwater sponge species (order Spongillida). Abbreviations: le, lentic; lo, lotic; ne, light negative; po, light positive; ac, acidic; ak, alkaline; lo, low; m-h, moderate to high; h, high. Modified from Harrison (1988a).

## Additional Information and Declarations

### Data Availability

The following information was supplied regarding data availability: This literature review does not include any raw data or code.
