# Supplementary material for: Utilizing sponge spicules in taxonomic, ecological and environmental reconstructions: a review
Source: PeerJ. 2020 Dec 18;8:e10601. doi: 10.7717/peerj.10601 (PMC7751429; doi:10.7717/peerj.10601)
Supplement: Supplemental Information 1 [file peerj-08-10601-s001.docx]

**Supplementary Information 1**

The list of the most relevant articles dealing with the application of sponge skeletal elements (spicules) in taxonomic, ecological, and environmental studies.

The list of references includes only those articles that are not cited in the main text.

| **Year** | **Language of publication** | **Publication** | **Fresh waterterr** | **Marine** | **Parameters** | **Locality** | **Age** |
| --- | --- | --- | --- | --- | --- | --- | --- |
| 1892 | English | Hinde & Holmes |  | x | Taxonomy, water depth | New Zealand | Eocene |
| 1894 | German | TraxlerReaxlerTTTTTT Traxler | x |  | Taxonomy | Europe | Miocene |
| 1910 | English | Hinde |  | x | Taxonomy | Southern Australia | Eocene |
| 1961 | Russian | Koltun |  | x | Taxonomy, ecology, water depth | N Ural | Cetaceous and Paleogene |
| 1963 | English | Jones & Beavers | x |  | Soil origin | Illinois, USA | - |
| 1966 | English | Racek | x |  | Taxonomy | Northern Guatemala | Up to 30,000 YBP |
| 1968 | English | Cavaroc & Ferm |  | x | Shoreline reconstruction | NE USA | Pennsylvanian |
| 1968 | English | Wilding & Drees | x |  | Soil origin | Ohio, USA | - |
| 1970 | English | Racek | x |  | Taxonomy and ecology | Italy | 30,000 YBP |
| 1974 | English | Racek | x |  | Taxonomy and ecology | Israel | Quaternary |
| 1978 | Polish | Moczydłowska & Paruch-Kulczycka |  | x | Taxonomy | Central S Poland | Late Jurassic |
| 1979 | English | Harrison et al. | x |  | Water alkalinity, regime, eutrophication | Florida, USA | Up to 4,000 YBP |
| 1980 | English | Hall & Herrmann | x |  | Taxonomy, water hardness (including Ca and Mg level), alkalinity | Colorado, USA | Up to 6,680 YBP |
| 1984 | English | Inoue |  | x | Taxonomy | Sagami Bay, Japan | Surface sediment |
| 1985 | English | Inoue |  | x | Taxonomy | Sagami Bay, Japan | Surface sediment |
| 1985 | English | Turner | x |  | Water alkalinity, SiO2 content, oxygenation, mineralization | West USA | 115,000 - 11,700 YBP |
| 1985 | English | Williams | x |  | Taxonomy, water temperature, alkalinity, pH, mineralization | Northern Idaho, USA | Miocene |
| 1986 | English | Harrison & Warner | x |  | Taxonomy, water depth, regime, pH, conductance | W Canada | 9,400 and 8,500 YBP |
| 1987 | English | Palmer | x |  | Water depth | Bahamas, Caribbean | Oligocene, Miocene |
| 1989 | English | Wagoner et al. |  | x | Sea level fluctuations | Canadian Ice Island | Modern |
| 1991 | English | Kratz et al. |  | x | Dissolved reactive silica concentrations | Wisconsin, USA | Up to ~12,000 YBP |
| 1991 | English | Molina-Cruz et al. |  | x | Opal accumulation | Iceland Sea | Holocene |
| 1991 | English | Wilkins et al. |  |  | Water depth | Kentucky, USA | 20,000 YBP |
| 1992 | English | Volkmer-Ribeiro | x |  | Marshland indicators | Brazil | Surface sediments |
| 1993 | English | Cumming et al. | x |  | Water salinity | W Canada | Surface sediments |
| 1993 | English | Yang et al. | x |  | Water quality and pollution | SE Canada | Last 330 Y (before 1993) |
| 1994 | English | Schwandes & Collins | x |  | Soil origin | Florida, USA | - |
| 1994 | English | Wiedenmayer |  | x | Taxonomy, paleogeography, ecology | Worldwide | Post Palaeozoic |
| 1995 | English | Robinson and Hasieit |  | x | Water depth | Ecuador | Mid-Early Miocene |
| 1995 | Portuguese | Volkmer-Ribeiro & Motta | x |  | Taxonomy | Central brazil | Surface sediment |
| 1996 | English | Volkmer-Ribeiro & Turcq | x |  | Weather patterns | Central Brazil | 27,500 - 51,780 YBP |
| 1997 | English | Paduano & Fell | x |  | Taxonomy, community variations | Connecticut, USA | Up to ≤1875 |
| 1997 | English | Mehl & Lehnert |  | x | Sedimentary environment | N Argentine | ?Cambro-Ordovician |
| 2000 | English | Candido et al. | x |  | Taxonomy, lake history | N Brazil | Up to 4,700 YBP |
| 2000 | English | Gammon et al. |  | x | Climate | Southern Australia | Eocene |
| 2001 | English | Ehrenberg et al. |  | x | Water depth variations | Spitsbergen | Perm/Triassic boundary |
| 2001 | Russian | Weinberg | x |  | Taxonomy, climate, water temperature | Southern Siberia, Russia | Late Pliocene |
| 2002 | English | Jach |  | x | Water depth variations | Tatra Mountains | Lower Jurassic |
| 2003 | English | De La Rocha et al. |  | x | Si concentration in water | Maud Rise, Antarctica (Southern Ocean) | Eocene-Oligocene boundary |
| 2003 | English | **Pisera** & Saez | x |  | Water pH, depth | N Chile | Miocene |
| 2004 | English | Gaiser et al. | x |  | Hydrological history | South Carolina, USA | Holocene |
| 2006 | English | Volkmer-Ribeiro et al. | x |  | Sediment type, water depth and characteristics, water level changes prediction | Southern Brazil | Surface sediments |
| 2006 | English | Pisera et al. |  | x | Taxonomy, water depth | Portugal | Miocene |
| 2006 | English | Elwood et al. |  | x | Ge and Si concentrations in water | Worldwide | Surface sediments and up to ~170,000 YBP |
| 2007 | English | Parolin et al. | x |  | Water regime, climate | S Brazil | 4,000 - 11,000 YBP |
| 2007 | English | Volkmer-Ribeiro et al. | x |  | Environment type | Eastern Argentine | Up to 11,000 YBP |
| 2007 | English | Volkmer-Ribeiro and Machado | x |  | Taxonomy | E Brazil | Surface sediments |
| 2008 | English | Parolin et al. | x |  | Water regime, climate | Central-western Brazil | 4,350 - 32,740 YBP |
| 2008 | English | Schindler et al. | x |  | Fresh water environment indication | France, Germany | Permo-Carboniferous |
| 2009 | Portuguese | Almeida et al. | x |  | Sponge community variations | East Brazil | Late Pleistocene - Holocene |
| 2009 | English | Mazzullo et al. |  | x | Water energy, water depth | South-central Kansas, USA | Lower Carboniferous |
| 2010b | English | Hendry et al. |  | x | Si concentration in water | Southern Ocean | 26,500 - 19,000 YBP |
| 2012 | English | Bertolino et el. |  | x | Sponge community stability | Ligurian Sea, Mediterranean | 100 - 200 Y |
| 2012 | English | Gaino et al. | x |  | Water temperature | Central Italy | Up to150 YBP |
| 2012 | English | Machado et al. | x |  | Water regime, taxonomy | Central Brazil | 39,700 YBP |
| 2012 | English | McGlue et al. | x |  | Water level changes | Central Brazil | Holocene |
| 2012 | English | Jochum et al. |  | x | Water temperature | East China Sea | 11,000 YBP |
| 2012 | Portuguese | Silva et al. | x | x | Water depth variations, sea water transgressions | South-east Brazil | Up to 6,240 YBP |
| 2013 | English | Guerreiro et al. | x |  | Climate | NW Brazil | Holocene |
| 2013 | English | Łukowiak et al. |  | x | Taxonomy | Panama | Surface sediments |
| 2013 | English | Kuerten et al. | x |  | Water regime | NW Brazil | Holocene |
| 2013 | English | Pisera et al. | x |  | Climate | N Canada | Eocene |
| 2014 | English | Bertolino et al. |  | x | Taxonomy | Mediterranean Sea, Ligurian Sea |  |
| 2014 | English | Machado et al. | x |  | Evolution of limnic system including climate | Central-west Brazil | 27,500 to more than 51,780 YBP |
| 2014 | English | Frisone et al. |  | x | Sedimentary environment | N Italy | Eocene |
|  |  |  |  |  |  |  |  |
| 2014 | English | Łukowiak et al. |  | x | Taxonomy | Central Paratethys, Slovakia | Miocene |
| 2014 | English | Ritterbush et al. |  | x |  | Eastern Panthalassa | Triassic/Jurassic |
| 2015 | English | Łukowiak |  | x | Taxonomy | S Australia | Eocene |
| 2016 | English | Fontorbe et al. |  | x | Si concentration in water | North Atlantic | Paleogene |
| 2016a | English | Łukowiak |  | x | Paleobiogeography, water depth | Australia | Eocene |
| 2016b | English | Łukowiak |  | x | Taxonomy | Panama | Surface sediments |
| 2016 | English | Murillo et al. |  | x | History of the sponge group (Geodiidae) | Newfoundland,  Canada | Up to 17,000 YBP |
| 2016 | English | Machado et al. | x |  | Taxonomy, ephemerism of lake | Central western Brazil | Surface sediments |
| 2016 | English | Pisera et al. | x |  | Climate | N Canada | Eocene |
| 2016 | Portuguese | Santos et al. | x |  | Climate, water regime | North-eastern Brazil | 8,410 - 3,929 YBP |
| 2017a | English | Bertolino et al. |  | x | Sponge community variation | Mediterranean Sea, Ligurian Sea | Up to 6,000 YBP |
| 2017b | English | Bertolino et al. |  | x | Sponge community variations | Ionian Sea, Mediterranean | 6207 - 1767 YBP |
| 2017 | English | Jochum et al. |  | x | Si concentrations in waater | East China Sea | Up to 17,000 YBP |
| 2017 | English | Pronzato et al. | x |  | Paleobiogeography | Worldwide | Fossils up to late Carboniferous |
| 2017 | English | Tatzler et al. |  | x | Si concentration in seawater | China | Ediacaran-Cambrian transition |
| 2017 | English | Zviejkovski | x |  | Facies analysis (hydrach stages of island formation) | S Brazil | Up to 900 - 1000 YBP |
| 2018 | English | Van Duyl et al. |  | x | C & N isotopes – water properties and nutrition | N Caribbean Sea | Surface sediments |
| 2018 | English | Łukowiak et al. |  | x | Community variation, correlation with variations of other physical parameters and reef inhabitants | Panama | ~1000 YBP |
| 2018 | English | Rezende et al. | x |  | Changes in hydrological conditions in two lakes | Central-Southern Brazil | 13,160 and 19,850 YBP |
| 2019 | English | Bertolino et al. |  | x | Taxonomy | Mediterranean Sea (Tyrrhenian Sea) | 5,800 and 3,700 YBP |
| 2019 | English | Łukowiak et al. |  | x | Taxonomy | Ukraine | Eocene |
| 2019a | English | Rasbold et al. | x |  | Taxonomy, water regime, sedi- mentary environment, climate | S Brazil | Late Pleistocene and Holocene |
| 2019b | English | Rasbold et al. | x |  | Taxonomy, hydrology, climate | Western Brazil | Up to 19,000 YBP |
| 2020 | English | Dumont et al. |  | x | Silicon cycle variations | Southern Ocean | 12,900 - 11,700 YBP |
| 2020 | English | Matheson and Frank |  | x | Water oxygenation, water depth, energy and salinity | Western USA | Permian |

Almeida, A.C.S., Volkmer-Ribeiro, C., Varajão, A.F.D.C., Gomes, N.S., and Varajão, C.A.C. 2009. Espículas de esponjas continentais nos sedimentos Cenozóicos do noroeste de Minas Gerais, como indicadores paleoambientais*.* Revista Brasileira de Paleontologia 12(2): 123-138. <http://dx.doi.org/10.4072/rbp.2009.2.03>.

Candido, J.L., Volkmer-Ribeiro, C., Filho, F.L.S., Turcq, B.J., Desjardins, T., and Chauvel, A. 2000. Microsclere variations of *Dosilia pydanieli* (Porifera, Spongillidae) in Caracaranã Lake (Roraima–Brazil): Palaeoenvironmental implications. Biociências 2: 77–92.

Dumont, M., Pichevin, L., Geibert, W., Crosta, X., Michel, E., Moreton, S., Dobby, K., and Ganeshram, R. 2020. The nature of deep overturning and reconfiguration of the silicon cycle across the last deglaciation. Nature Communications 11: 1534. Doi: 10.1038/s41467-020-15101-6

Duyl, van, F.C., Mueller, B., and Meesters E.H. 2018. Spatio-temporal variation in stable isotope signatures (d13C and d15N) of sponges on the Saba Bank. PeerJ 6: e5460. DOI 10.7717/peerj.5460

Ehrenberg, S.N., Pickard, N.A.H., Henriksen, L.B., Svånå, T.A., Gutteridge, P., and Macdonald, D. 2001. A depositional and sequence stratigraphic model for coldwater, spiculitic strata based on the Kapp Starostin Formation (Permian) of Spitsbergen and equivalent deposits from the Barents Sea. Bulletin 85: 2061–2087. doi:10.1306/8626D347-173B-11D7-8645000102C1865D

Jach, R. 2002. Lower Jurassic spiculite series from the Križna Unit in the Western Tatra Mts, Western Carpathians, Poland. Annales Societatis Geologorum Poloniae 72: 131–144.

Matheson, E.J. and Frank, T.D. 2020. An epeiric glass ramp: Permian low-latitude neritic siliceous sponge colonization and its novel preservation (Phosphoria Rock Complex) Sedimentary Geology 399: 105568. https://doi.org/10.1016/j.sedgeo.2019.105568

Mazzullo, S.J., Wilhite, B.W., and Woolsey, I.W. 2009. Petroleum reservoirs within a spiculite-dominated depositional sequence: Cowley Formation (Mississippian: Lower Carboniferous), south-central Kansas. Bulletin 93: 1649–1689. doi:10.1306/06220909026

Mehl, D. and Lehnert, O. 1997. Cambro-Ordovician sponge spicule assemblages in the Ordovician of the Argentine Precordillera and paleoenvironmental ties. Jb. Geol. Palaont. Abh. 204: 221–246.

Murillo, F.J., Kenchington, E., Lawson, J.M., Li, G., and Piper, D.J.W. 2016. Ancient deep‑sea sponge grounds on the Flemish Cap and Grand Bank, northwest Atlantic. Mar Biol 163: 63. DOI 10.1007/s00227-016-2839-5

Paduano, G.M. and Fell, P.E. 1997. Spatial and temporal distribution of freshwater sponges in Connecticut lakes based upon analysis of siliceous spicules in dated sediment cores. Hydrobiologia 350: 105–121.

Palmer, A.A. 1987. Paleoenvironmental significance of siliceous sponge spicules from sites 627 and 628 , little Bahama Bank, ocean drilling program Leg 101. In Rose, W.D. and Stewart, S.K (eds) Proceedings of the Ocean drilling Program, Scientific Results, 101: 159–168, College Station ,Texas.

Rezende, A.B., Stevaux, J.C., Parolin, M., and Guerreiro, R.L. 2018. Sponge spicules as proxy in Upper Quaternary lake deposits near Cianorte (PR): a tentative correlation with lakes of Central-Southern Brazil (NW Paraná and SE Mato Grosso do Sul). Quaternary and Environmental Geoscience 9(2): 10–18.

Ritterbush, K.A., Bottjer, D.J., Corsetti, F.A., and Rosas, S. 2014. New evidence on the role of siliceous sponges in ecology and sedimentary facies development in eastern Panthalassa following the Triassic-Jurassic mass extinction. Palaios 29: 652–668. doi:10.2110/palo.2013.121

Robinson, P.D. and Hasieit, S.K. 1995. A radiolarian dated sponge microsciere assemblage from the Miocene DOS Bocas Formation of Ecuador. Journal of South American Earth Sciences 8(2): 195–200.

Silva, K.C., Parolin, M., and Bissa, W.M. 2012. Espículas de esponjas *vs.* variações do nível relativo do mar na região de iguape, sudeste do Brasil. Rev. Bras. Paleontol. 15(3): 319–326. doi: 10.4072/rbp.2012.3.xx

Tatzel, M., Blanckenburg, F. von, Oelze, M., Bouchez, J., and Hipple, D. 2017. Late Neoproterozoic seawater oxygenation by siliceous sponges. Nature communications 8: 621. Doi: 10.1038/s41467-017-00586-5

Volkmer-Ribeiro, C. and Motta, J.F.M. 1995. Esponjas formadoras de espongilitos em lagoas no triângulo mineiro e adjacências,com indicação de preservação de habitat. Biociências 2: 145–169.

Wagoner, N.A. van, Mudie, P.J., Cole, F.E., and Daborn, G. 1989. Siliceous sponge communities, biological zonation, and Recent sea-level change on the Arctic margin: Ice Island results Canadian Journal of Earth Sciences 26(11): 2341–2355.

Weinberg, E.V. 2001. The sponge fauna of lake Baikal in the Late Pliocene. Russ Geol Geophys. 1: 130–137. [In Russian]

Williams, J.L. 1985. Spicular remains of freshwater sponges from a Miocene lacustrine deposit in Northern Idaho. In: C.J. Smiley, A.E. Leviton, and M. Berson (eds.), Late Cenozoic History of the Pacific Northwes*t*, pp 349–355. Allen Press, Lawrence.
