# Supplementary material for: Utilizing sponge spicules in taxonomic, ecological and environmental reconstructions: a review
Source: PeerJ. 2020 Dec 18;8:e10601. doi: 10.7717/peerj.10601 (PMC7751429; doi:10.7717/peerj.10601)
Supplement: Supplemental Information 2 — Abbreviations: le, lentic; lo, lotic; ne, light negative; po, light positive; ac, acidic; ak, alkaline; lo, low; m-h, moderate to high; h, high. Modified from Harrison (1988a). [file peerj-08-10601-s002.docx]

**Supplementary Information 2**

Environmental preferences of selected freshwater sponge species (order Spongillida).

Abbreviations: le. – lentic, lo. – lotic, ne. – light negative, po. – light positive, ac. – acidic, ak. – alkaline, lo. – low, m-h. – moderate to high, h. – high. Modified from Harrison (1988a).

| Sponge species | Water regime | Light intensity | pH values | Values of bicarbonate ions | Differing conductivity values | Values of Ca in the medium |
| --- | --- | --- | --- | --- | --- | --- |
| *Anheteromeyenia ryderi* | le | ne |  | lo |  | lo |
| *Anheteromeyenia argyrosperrna* | lo | po | ac | lo | h |  |
| *Anheteromeyenia pictovensis* |  | ne |  |  |  |  |
| *Anheteromeyenia ryderi* |  |  | ac |  |  |  |
| *Corvomeyenia carolinensis* | le |  |  |  |  |  |
| *Corvomeyenia everetti* |  |  | ac | lo | lo | lo |
| *Dosilia radiospiculata* |  |  | ak | mo-h | h |  |
| *Ephydatia fluviatilis* | lo | ne |  | mo-h | h |  |
| *Ephydatia robusta* |  |  | ac |  |  |  |
| *Eunapius fragilis* | lo | ne |  | mo-h | h |  |
| *Eunapius mackayi* |  | ne | ac |  | lo | lo |
| *Ephydatia mulleri* | lo | ne |  | mo-h | h |  |
| *Heteromeyenia latitenta* | lo |  |  |  |  |  |
| *Heteromeyenia tubisperma* | lo | po |  | mo-h |  |  |
| *Heteromeyenia baileyi* | le |  |  |  |  |  |
| *Heteromeyenia tentasperma* | le |  |  | mo-h |  |  |
| *Radiospongilla crateriformis* | le |  | ak | mo-h | h |  |
| *Spongilla aspinosa* | le |  | ac |  |  |  |
| *Spongilla cenota* | le |  | ak |  |  | h |
| *Spongilla lacustris* | lo | po | ak | lo | h |  |
| *Spongilla wagneri* | lo |  |  |  |  |  |
| *Trochospongilla horrida* |  | ne | ak | mo-h |  | h |
| *Trochospongilla leidyi* |  | ne | ak | mo-h | h | lo |
| *Trochospongilla pennsylvanica* | le |  | av | lo | lo | lo |
